# How Do Health Systems Address Patient Flow When Services Are Misaligned With Population Needs? A Qualitative Study

**DOI:** 10.34172/ijhpm.2021.36

**Published:** 2021-04-26

**Authors:** Sara Kreindler, Zaid Aboud, Stephanie Hastings, Shannon Winters, Keir Johnson, Sara Mallinson, Meaghan Brierley

**Affiliations:** ^1^Department of Community Health Sciences, Max Rady College of Medicine, University of Manitoba, Winnipeg, MB, Canada.; ^2^George & Fay Yee Centre for Healthcare Innovation, Winnipeg Regional Health Authority/University of Manitoba, Winnipeg, MB, Canada.; ^3^Health Systems Evaluation & Evidence, Alberta Health Services, Calgary, AB, Canada.

**Keywords:** Patient Access, Health System Organization, Emergency Department Crowding, Qualitative Research, Canada

## Abstract

**Background:** Patient flow through health services is increasingly recognized as a system issue, yet the flow literature has focused overwhelmingly on localized interventions, with limited examination of system-level causes or remedies. Research suggests that intractable flow problems may reflect a basic misalignment between service offerings and population needs, requiring fundamental system redesign. However, little is known about health systems’ approaches to population–capacity misalignment, and guidance for system redesign remains underdeveloped.

**Methods:** This qualitative study, part of a broader investigation of patient flow in urban Western Canada, explored health-system strategies to address or prevent population–capacity misalignment. We conducted in-depth interviews with a purposive sample of managers in 10 jurisdictions across 4 provinces (N = 300), spanning all healthcare sectors and levels of management. We used the constant comparative method to develop an understanding of relevant strategies and derive principles for system design.

**Results:** All regions showed evidence of pervasive population–capacity misalignment. The most superficial level of response – mutual accommodation (case-by-case problem solving) – was most prevalent; capacity (re)allocation occurred less frequently; population redefinition most rarely. Participants’ insights yielded a general principle: Define populations on the basis of clusters of co-occurring need. However, defining such clusters demands a difficult balance between narrowness/rigidity and breadth/flexibility. Deeper analysis suggested a further principle: Populations that can be divided into homogeneous subgroups experiencing similar needs (eg, surgical patients) are best served by narrow/ rigid models; heterogeneous populations featuring diverse constellations of need (eg, frail older adults) require broad/ flexible models.

**Conclusion:** To remedy population–capacity misalignment, health system planners should determine whether clusters of population need are separable vs. fused, select an appropriate service model for each population, allocate sufficient capacity, and only then promote mutual accommodation to address exceptions. Overreliance on case-by-case solutions to systemic problems ensures the persistence of population–capacity misalignment.

## Background

**Key Messages**

**Implications for policy makers**

- Population–capacity mismatches were pervasive across all health jurisdictions studied. Of the three levels of response to such misalignment, the most superficial (case-by-case problem solving) was most prevalent, with less attention given to capacity (re)allocation and almost none to population definition.
- This is the reverse of the order that should be adopted. Health system planners should first determine population needs, and then allocate capacity, using mutual accommodation only to address a limited number of exceptions.
- While there is agreement that populations should be defined in terms of clusters of co-occurring need, trade-offs are involved in determining how narrowly/rigidly or broadly/flexibly these clusters should be defined.
- A useful principle for managing these trade-offs is that relatively narrow/rigid definitions work for homogenous populations with similar needs (eg, surgical patients); while heterogeneous populations with diverse constellations of need (eg, frail elderly) require broad/flexible definitions.
- We outline the features of appropriate service models for populations in which clusters of need are clearly separable (segmentation, specified services, sorting rules) vs. inevitably fused (fuzzy sets, fluid resources, funnels).

**Implications for the public**

All countries are working to improve the movement of patients through the health system so that they can obtain the care they need. Patients cannot move smoothly through the system unless available services are a good match for their needs. When service offerings do not match population needs, health system planners should first define all populations in need of care, then assign appropriate capacity. In practice, however, we found that mismatches were usually addressed by case-by-case problem solving, seldom by ensuring that services are first designed to fit population needs. This results in system inefficiency and unmet need. Defining the populations in need of care is a complex task involving many trade-offs. However, our findings suggest a guiding principle: Use relatively narrow/rigid definitions when it’s possible to identify distinct subgroups with similar needs (eg, surgical patients), and broad/flexible definitions when needs are complex and overlapping (eg, services for seniors).

Patient flow – ensuring that patients can move smoothly through the health system to obtain the care they need – is one of the greatest challenges facing healthcare today. The issue of flow is vast, intersecting with multiple dimensions of healthcare quality,^1^ and is a theme of myriad improvement efforts in diverse areas. While emergency department (ED) crowding is its most recognizable symptom, stagnant flow may result from bottlenecks at any point along the continuum of care^2^; as such, flow is widely recognized as a system problem that demands a system response. However, existing evidence is inadequate to guide decision-makers through this difficult, complex and potentially risky undertaking.

 The literature has focused overwhelmingly on *specific interventions* to reduce input, accelerate throughput, or facilitate output from the ED or hospital.^3-5^ However, it seems doubtful that such interventions hold the key to improving flow. The overall evidence base for flow interventions is weak: Although virtually every type of initiative has shown some promising results, very few can be confirmed as effective, considering the methodological shortcomings of the available studies and the high likelihood of publication bias.^6,7^ Furthermore, no specific intervention, nor combination thereof, has been found to predict flow performance at the hospital level, and health systems with very poor performance may nonetheless boast a plethora of interventions.^9^ A large systematic review suggested that interventions are often poorly matched to the actual causes of flow problems, noting that greater organizational and scholarly attention has been devoted to testing solutions than to understanding problems.^10^ Moreover, when the underlying problem exists at the system level, it is unlikely to be amenable to localized solutions.^9^ Improving the efficiency of one department within an interdependent health system does not necessarily improve system throughput, and may even have the unintended consequence of creating bottlenecks.^11^ There has been growing interest in “whole-of-system” approaches to flow,^10^ as reflected in research on system-level leadership/management practices (eg, data-driven management, performance accountability^8^) and system-wide strategies (eg, enforcement of ED length-of-stay targets^12,13^) that may improve flow performance.

 However, to date there has been limited attention to the issue of system *design*. This gap is critical, because if the system is not designed to provide “the right care to the right patients,” it is highly unlikely to be able to provide care at “the right time.” The theme of system design pervades the theoretical literature on flow: The Theory of Constraints^14^ and the Theory of Swift Even Flow^15^ emphasize the need for system-level re-engineering to address the bottlenecks and sources of variability that impede the smooth flow of people or materials through a process. Such theory, derived from the field of operations management, has had an indelible influence on researchers’ understanding of healthcare wait times and flow.^16-18^ In practice, however, reengineering is usually applied to discrete parts of the healthcare system, not to the system as a whole.^11,19^ Looking beyond the flow literature, the general literature on delivery system redesign points to the need for new models of care to address shifting patterns of population need.^20^ In particular, it has long been recognized that many health systems, having been developed to address acute medical problems, are misaligned with growing needs for complex and continuing care.^21,22^ However, since such misalignment is not typically conceptualized as a *flow *issue, the implications for flow have received little examination.

 A recent explanatory case study yielded a model for conceptualizing system design in relation to patient flow: the population-capacity-process model.^9,23^ According to this model, patient flow depends on clearly identifying all populationsin need of care and directing each to suitable capacity through a streamlined, reliable process. Here, *population* refers not to a demographic group but to those who share a need or set of needs; defining a population by needs facilitates identification of the most appropriate *capacity* (physical and human resources) to meet those needs, and development of an efficient *process *whereby the intended patients can access this capacity.^9^ The study, which probed the source of one health system’s intractable flow problems, identified a fundamental misalignment between service offerings and population needs.^23^ Symptoms of such *population–capacity misalignment *included frequent identification of patients for whom no suitable service existed, habitual assignment of patients to a suboptimal service because the appropriate one was full (eg, “off-servicing”), and conflict between programs and/or sites as to who should take responsibility for certain categories of patient. As the underlying problem existed at the level of system design, even evidence-informed, well-managed flow initiatives fell far short of their desired impacts. Moreover, the failure of specific flow interventions could be linked to their neglect of one or more of the three domains: population, capacity, and/or process.^9^ Research in other organizations has corroborated these findings at the micro (initiative) level.^24,25^ At the macro level, however, there is a need to explore the potential problem of population–capacity misalignment in other health systems, not limited to exceptionally poor performers.

 One might suppose that most systems avoid misalignment through periodic assessment and/or forecasting of population needs and incorporation of this information into the planning cycle. Indeed, many countries and provinces use forecasting to inform workforce planning^26^; many hospitals and health systems conduct community health needs assessments^27,28^; and some use mathematical modelling to guide the distribution of physical capacity.^29,30^ However, prevailing approaches to forecasting may be of limited value in helping systems adapt to changing needs, as models typically factor in only demographic trends and current patterns of utilization, not shifts in epidemiology or provider productivity.^26^ Meanwhile, health system planners may not take action on the findings of needs assessments, or may respond with superficial changes.^27,31^ To the extent that planning processes are biased towards the reproduction of existing arrangements, they may fail to correct misalignment.

 This qualitative study undertook an open-ended exploration of how healthcare managers engage with the issue of population–capacity (mis)alignment. The research questions were:

1. How do health systems address and/or avoid population–capacity misalignment?
2. What principles can be derived for optimal system design?

## Methods

###  Study Design and Context

 This study was a core component of a multi-jurisdictional research project designed to explore in depth how health systems can achieve maximal improvement in patient flow. The Western Canadian Flow (WeCanFlow) study, which spanned the 10 urban health regions/zones of Canada’s four western provinces, was designed in partnership with decision-makers from all participating jurisdictions and funded by the Canadian Institutes of Health Research. In keeping with an integrated knowledge translation approach, decision-makers were involved in identifying relevant research questions, giving feedback on recruitment materials and interview guides, identifying potential participants, and reviewing and interpreting findings.

 In Canada, health is a provincial/territorial responsibility; within many provinces, regionally based bodies (eg, health authorities) are responsible for the funding and delivery of hospital, community, and long-term care, as well as mental and public health services. The participating regions were disparate in size, structure, history, and political context,^32^ affording an ideal opportunity to explore system-level issues.

 The overall project was originally conceived as a mixed-methods comparative case study; its objective was to quantitatively assess inter-regional differences in flow performance and qualitatively explore factors differentiating high from low performers. System design was chief among the factors of interest; other factors included specific flow initiatives and aspects of social/organizational context. However, quantitative analysis revealed that inter-regional performance variation was not sufficiently large or consistent to permit comparison of performance-based subgroups. As preliminary qualitative analysis also suggested more cross-jurisdictional similarities than differences, we decided to treat the qualitative dataset as a whole rather than as ten separate cases (while of course remaining attentive to any regional variation).

 Approval was obtained from all relevant bodies for ethical and operational review in Manitoba, Alberta, Saskatchewan, and British Columbia, including one university (harmonized) research ethics board per province and the operational review committee of each participating region. All participants signed a consent form.

###  Data Collection

 The primary data source consisted of in-depth interviews with 300 managers and quality improvement staff with current or recent responsibility for patient flow and/or involvement in flow initiatives. Participants included ~20-45 purposively sampled personnel from each region and 5 from extra-regional bodies such as provincial ministries and quality councils. We sought representation of different levels of management (regional, organizational, departmental, etc) and organizations (eg, hospitals, programs), and a mix of strategic and operational roles. In keeping with our understanding of patient flow as an issue that spans the continuum of care, we recruited participants from all sectors, not only acute care. The sample was 60% female and predominantly at (39%) or above (45%) the Director level.

 Potential participants were identified from organizational charts and by key decision-makers. Email addresses were gathered by a member of the participant’s own region (researcher or decision-maker) who had access to the organizational directory, and were not shared with the full team. The local member sent each recruitee a personalized email introducing the study and inviting them to contact the research coordinator; reminders were sent 2-3 weeks later if necessary. A few additional participants were identified through snowball sampling. The overall response rate was 67%; region-specific rates ranged from 35%-88%.

 Most of the interviews, typically 45-60 minutes in length, were conducted in person at the participant’s office or a location of their choice; telephone was used for individuals who were unavailable during the site visit, and for one region where the site visit was cancelled due to weather. While most interviews were individual, in a few cases 2-3 colleagues chose to participate together.

 Interviews were conducted between April 2016 and April 2018 by a trained, Master’s or PhD-prepared interviewer. All interviewers were or had recently been embedded researchers in one of the participating regions; as such, they had experience working with decision-makers and were known to some participants, but were not in a supervisory role over any. Participants were aware that the research was intended to help Western urban health regions improve flow across the continuum of care, and that decision-makers planned to use the findings.

 The semi-structured interview guide included questions about flow strategies, system design and organizational context, including participants’ current and past involvement in flow initiatives, and perspectives on what had (not) worked well. Interviews were audio-recorded and transcribed. For the broader study, interview data were supplemented by document review and non-participant observation of flow meetings/events, but these methods did not contribute to the present sub-study except as a source of contextual information.

###  Data Analysis 

 Interview data were entered in NVivo 11. In a preliminary round of analysis (“bucket coding”), two researchers content-analyzed each interview independently to identify extracts related to the issue of population–capacity (mis)alignment; disagreements were resolved by consensus. Selected extracts were analyzed using the constant comparative method.^33^ Themes were identified inductively; then, codes were (re)categorized and elaborated using the population-capacity-process model as a sensitizing concept. Analysis relied on an iterative process of constant comparison between extracted quotations, full transcripts, contextual information, relevant literature, and the evolving coding scheme; this was led by one researcher with frequent team discussion of emerging interpretations. Contradictions in the dataset were explored through the technique of dialectical analysis^23^: when a disagreement was identified, we sought to ascertain the axis of conflict, then identify a principle that could synthesize two opposing viewpoints. All authors reviewed an initial draft report; as an additional measure to ensure trustworthiness, a revised draft was circulated to the full study team (comprising 31 health-system decision-makers and 17 researchers), with a request for review and commentary.

## Results

###  Symptoms of Population–Capacity Misalignment 

 All of the above-noted symptoms of population–capacity misalignment were reported in all regions (see Table 1). “Off-servicing” (admitting patients to beds intended for a different population) was widely practiced despite its recognized limitations; off-servicing from medicine into surgery was most common. Participants in each region reported difficulty finding suitable programs for complex high-needs clients, particularly those with psychosocial/behavioural issues. Participants also described conflict surrounding the eligibility criteria of both acute and community programs, although several noted that overt conflict had decreased over time. Overall, the theme of population­–capacity misalignment as a problem requiring ongoing management emerged from the great majority of interviews.

**Table 1:** Symptoms of Population–Capacity Misalignment

**Symptoms**	**Exemplar Quotes**
Pervasive off-servicing	*" So what we’re going to do is we’re going to get a high number of admissions and we’re going to load our OR and we’re going to off-service a whole bunch of patients to surgery and then lo and behold; the next day we’re going to be completely screwed because now there’s nine the next day, but I’ve off-serviced more medical and I imagine by the next day we’re even in worse shape" *(P7208). *"So, yeah, there's always off-servicing … I mean, if anybody is fighting that, they've been fighting that for years and years and years, because it's just a reality that we live in" *(P2109).
No place for patients with complex needs	*"Part of the challenge we face at this site is there’s some real complex patients that there isn’t a service [for] right now*" (P5217). *"And in order for me to get, say, your mother out of the hospital, what if your mother has a major history of bipolar depression and now she's had a head injury? Oh, this isn't mental health; sorry, she doesn't meet our criteria. So we talk to brain injury: we need brain injury housing. No, no, no; she's not a very good functioning bipolar patient; sorry. Okay, well, let's send her to a nursing home. No, no; [she's] only 53, she doesn't meet criteria" *(P2108).
Conflict	*“ … everybody wants to take over our service, everybody wants to run our service to suit their needs, and it is a constant struggle … And we do our best to, well, quite frankly ignore it … [or] I will tell you exactly what would happen: we would have a rehabilitation ward filled with long-term care patients that aren't appropriate for a rehabilitation service"* (P8112). *" So I think no matter what criteria you have … you're never going to have the perfect criteria, and because of that we always have conflicts and we continue to have conflicts" *(P10105).

###  Strategies for Addressing Population–Capacity Misalignment

 Analysis of actions taken or proposed by participants suggest three main categories of strategies used to manage population–capacity mismatch in the face of these challenges: *mutual accommodation *(case-by-case problem-solving); adding or repurposing *capacity* to better match observed need; and *redefining patient populations. *

 Mutual accommodation – finding ways to accommodate needs of individual patients without changing the design or allocation of capacity – emerged as the most common strategy. Not only was it reported to occur *ad hoc*, it was institutionalized through such initiatives as bed meetings (at which staff negotiated the distribution of new admissions), overcapacity protocols (which mandated the use of off-service and/or unfunded beds), and regular meetings between acute and nonacute personnel to determine discharge destinations for patients with complex needs. Every region had implemented at least one (typically several) such interventions, and the majority of participants mentioned their role in easing flow. Some expressed pride that staff collaborated to accommodate patients, though many recognized such processes as an unfortunate “work-around.”

*“In the in-patient world we have no choice: every bed is a space where we can put a patient. And until we design hospitals that are [at] 85% capacity we’re always going to run into this trouble … Yeah, these are all work-arounds. And it’s always not as good as it could be”* (P10130).

 While mutual accommodation was sometimes used to compensate for the lack of any program for which the patient was eligible, more commonly a suitable program existed but was fully subscribed, necessitating a “*bed shuffle*” (P4104). The daily “*juggling*” (P2202) of beds was often described as time-consuming and frustrating for managers and staff. Mutual accommodation strategies were also associated with risks (eg, off-servicing medical to surgical may result in cancellations to the surgical slate, or in the provision of suboptimal care) which required mitigation strategies (eg, protection of the surgical slate). These issues are discussed in greater depth in a separate paper on interventions for overcapacity management. Outside the acute sector, the process of mutual accommodation was less frenetic but still time-consuming and potentially frustrating.

*“It seems like we have to go through a lot of work before people actually come together and have a conversation and a meeting … to problem-solve, versus sitting in your office with an email and knowing that you’re going to have to go back to your team and explain why you’re okay with this person who doesn’t fit the mandate coming in and, ‘Did you lose the meeting? Is that what happened?’”* (P2104).

 Few participants spoke of attempting to eliminate the need for frequent mutual accommodation; there appeared to be an assumption that the system would/could not be designed to ensure that service offerings were closely aligned to patterns of population need.

 The second category of strategies, adding or repurposing capacity to better match the observed distribution of patient needs (see Table 2), was also well-reflected in every region. Each province had expressed a policy commitment to “shift care into the community,” and participants from all regions reported certain investments in postacute, transitional and/or home-based care. Typically, capacity-based strategies entailed expansion of already-existing services, although in a few cases capacity had been developed for newly identified populations (eg, persons with substance-abuse issues). Realignment of the hospital “bed map” to “right-size departments” was also reported in most regions, and undertaken regularly by at least two.

**Table 2:** Capacity (Re)Allocation Strategies

**Strategies**	**Exemplar Quotes**
Increasing capacity of services	*"The mental health population has exploded in children and youth … We’re constantly looking at capacity and how can we change anything and you know, tweak any kind of – anything … But we know we need more beds” *(P5225).
New services for underserved populations	*"And we recognize now that some of our alternate level of care patients; there are three populations that are hard to place: complex behaviours are very hard to place; ventilated patients are hard to place and bariatric patients are hard to place. So we need to build that capacity"* (P8106).
Redrawing bed map	*"We did a bed map analysis a couple of years ago … and we’re just in the process now of doing that again to say: where are we; does surgery have the beds they need; does medicine have the beds they need based on the patient population?" *(P9101).

 Although capacity-based strategies were within the repertoire of all regions, they were subject to fiscal and logistical constraints. Across jurisdictions, participants desired more capacity expansions than their region had been able to fund, and reallocation of resources was perceived as challenging (“*our biggest challenge is … yes, we wanna support people at home, but it does come at a cost, and we find that the cost just never comes from acute … because acute has their own issues*” [P1215]).

 The least prevalent strategy consisted of redefining populations in order to allocate capacity and designing processes accordingly. Although over one-third of participants contributed data related to population (re)definition, only a handful could point to examples in which it had actually occurred. The common theme of these few success stories was the combination of atomized programs in order to better serve patients.

 “*I had this many mobile teams … little pockets of staff: all working during the same hours; all tripping over each other and arguing about, ‘That’s your patient, not my patient’ … Whatever possessed us to staff 14 teams? … Great, now we can get rid of some of the silly structures around what we do” *(P9205).

*“The heart health value stream includes an inpatient unit; the CV surgery; it includes ECG tests, it includes the cath lab, it includes, it includes community resources. But they all see themselves as serving the heart health patient … It used to be very siloed and they’re all one now … and we do what’s best for our patient”* (P8103).

 Much more commonly, participants reported that program boundaries reflected historical conventions and resource availability, not deliberate design on the basis of population need. Several participants noted that criteria were kept narrow and rigid in order to protect scarce capacity and were relaxed when additional capacity became available.

*“Eligibility criteria in my mind is all about scarce resources. So we create eligibility criteria because we don’t have enough resources to meet that need. But every time you create eligibility criteria you almost always create a gap … But the challenge is, [we] just don’t have the capacity” *(P1104).

*“We got funded to do this, and then a few years later we got funded to do that, and by the way, don’t mix the two or you’ll lose your funding”* (P9205).

 Although positive examples were rare, many participants offered guidance on population redefinition by critiquing existing arrangements or exploring hypothetical scenarios. As their comments focused on what should be avoided in defining populations, we have termed the emerging themes “The Five Don’ts”: Do not (1) separate similar needs, (2) separate needs that occur together, (3) use arbitrary cut-off points, (4) separate needs closely sequenced in time, (5) combine dissimilar needs (see Table 3 for examples of each principle as identified by participants).

 These negative principles can be synthesized into one broader, positive principle: Define populations in terms of co-occurring need; that is, group patients who need essentially the same human and physical resources even if their characteristics differ in other ways.

**Table 3:** Principles for Redefining Populations: The 5 Don’ts

**Principle**	**Exemplar Quotes**
Do not separate similar needs (eg, acquired brain injury, dementia with aggression)	*"One of our biggest problems right now that we have no solution to is the patients that don’t fit any category … so I'll give you an example. Elderly client with a dementia, and element of aggressive behaviour. They don’t fit in residential care because they can be harmful to themselves and to others. They don’t fit in mental health because … they don’t have a mental health issue … We have acquired brain injury which is for a select group of patients, and these people don’t qualify. So they sit in our acute care system … we're beginning to have the dialogue now to say, we should be focusing on what people's needs are and not what their diagnosis is, because when we look at, for example, acquired brain injury a lot of what is done in that program could really be applicable to all of these other types of patients. … That's, I think, where we want to go, more based on patient need rather than the diagnosis” *(P3101).
Do not separate needs that frequently co-occur (eg, mental health, addictions)	*"Out of [the] people that we're seeing, about 20 to 25 percent would be a straight intoxication kind of thing, and about 20 to 25 percent are just pure psychiatric. The rest are drug and psychiatric, so about 60%. And many mental health teams still [say], 'No no, that's drugs. We don’t deal with drugs.' … they don't have an in-house addiction team. It's like – really?"* (P1101).
Do not use arbitrary cut-offs (eg, age)	*"[You can restrict a program to] the elderly population, older adults, so you’re not getting pediatric cases, you're not getting 24 years old … But 65 is an arbitrary number … If somebody is age 64 and they meet all your other criteria and you can help them … what are you going to say? Wait for another three months until you turn 65 and then I'll take you in?" *(P10136).
Do not separate needs that are closely sequenced in time (eg, services for frail seniors)	*" So Mrs. Jones comes in and she has dementia, she has pneumonia and she's fallen. And she goes to the acute bed. Then after, say, seven days she's been on antibiotics so her pneumonia is getting better, but functionally she has declined significantly based on being in bed. Then we send her to the sub-acute bed. And so a whole new team … So I believe that we were extending length of stay based on transferring people halfway through their course of illness. And I also believe that our units are not structurally or environmentally set up for people with dementia … So [instead] we'll have one unit that really focuses on the complex integrated: so [you] have an acute physical issue and [you] have … dementia … and instead of moving you from acute to sub-acute you're going to stay on one unit for your whole stay" *(P1111).
Do not combine dissimilar needs (eg, severe behavioural issues housed with vulnerable seniors)	*" So we have a lot of gang-related trauma that winds up being long term. So then there is no reasonable housing for those individuals. You can't put them with grandma in long term care, right? … So now we are working together with the organizations and say, how do we start to problem-solve these things?” *(P6106).

###  Trade-offs in System Design

 While there was broad consensus on the principle that services should be designed around clusters of co-occurring need, in practice the task of defining such clusters was experienced as challenging. Some participants made explicit reference to inherent trade-offs in defining populations, capacity, and process; at other times these trade-offs became apparent in comparing diverse perspectives on opposite sides of an apparent controversy. Underpinning each of the trade-offs (see Table 4) was found a common theme: the need to strike a balance between specificity and rigidity on the one hand, and breadth and flexibility on the other.

**Table 4:** Trade-offs in System Design

**Type of Trade-off**	**Exemplar Quotes**
Sensitivity-specificity trade-off *Issue: Defining eligibility criteria too broadly vs. too narrowly*	"*When [rehab] first became a program we had these broad, diffuse criteria, wanting to be of service [to] anyone who could benefit … The impact … we saw [was a lack of] impetus [for] acute care to do all the work to get the patient to go directly home … because there's this downstream place or safety net. … So it's a careful balance because there's pros and cons to criteria. You get too narrow and specific with criteria, in the absence of anywhere else for those patients to go, then they sit in acute care, which is not good. … But I get really worried about broad, diffuse criteria or the perception that, well, if you got empty beds you just take these folks, because … then the easy button, not necessarily the right button, is to dump them over there and let someone else deal with them" *(P3106). *"I get both sides. It's very hard. If you dilute your criteria over and over and over, not just on a really rare exceptional basis, then your program is no longer the program that it was set up to be or to serve the population that it was needed to serve … [But] we don’t have endless staff or programs. So then what do you do with those people [who don't fit]?" *(P2103).
Carve-out trade-off *Issue: Dividing service users into too few vs. too many groups*	*" So I think the evidence says that the more you can sub-specialize the more you can move people through the system quicker because you’ve got teams that really understand the patient’s needs and can treat them quickly. But the downside of that is, what about all the general patients and where do they go? And when you’ve got 24 trauma patients and only 18 trauma beds, where do these trauma patients go?" *(P5221). *"You know we have done a lot of breaking our clients up into such micro groups … we cut it too fine … [M] aybe [those models] did work well in [cities] that are much bigger centres … but here maybe we don’t have the overall [volume] of patients to meet the defined need, you know. It's like you can segregate patients down to the minutest amount but in the end you lose the ability to operate because it's so small and so restricted you can’t actually flow"* (P8109).
Bespoke trade-off *Issue: Undertaking too much vs. too little customization of services*	*"But as a system, should we be stretching criteria to squeeze people into it? Or should we be having more of a person- centred approach? … So program A is not a good fit, program B is not a good fit, program C – so we're going to go back to program A but we're going to stretch it so it has an element of program B" *(P10107). *"So go back two years ago with the high volumes of people waiting in Emergency, decisions were made that somebody has been [there] 100 hours, you need to find a place for them and a specialized contract will be put in place, and sometimes those were incredibly expensive. If you want something that fast that's individualized, not planned around a group need or around a population need, you're paying a pretty big dollar to get that in place … It would be like if you said people needed personal care homes and there was no space, so we'll simply pay for somebody to take care of this person, and we'll pay for somebody to take care of that person … but eventually you count and those ten people would have funded an entire personal care home. Okay – maybe we should fund the personal care home*" (P10110).
Sensitivity-specificity and bespoke trade-offs in interaction *Issue: Broadly defined populations cannot be served with narrowly-defined capacity*	*"Whenever you are moving patients to [a] lower acuity [unit], any experienced practitioner … knows the barriers you run into, because you go to move the patient and someone says, 'Yeah, no, we don’t run that drug on the unit.' … So … you can't have broad sweeping statements about what an area does. You can have a few broad sweeping statements, but unless it's sub-populated by 'specifically we will do this … we won't do that' … you will run into this endless loop forever … And you'll be re-inventing the wheel all along. … you just find yourself in the trap of, 'I gave you money to do something, you tell me you're doing it but you can't take the patients'"* (P10132).
Bottleneck trade-off: funnel vs. sorting models *Issue: Potentially creating bottlenecks at the point of assessment (funnel model) vs. the point of referral (sorting model)*	*"Over the next year...we want to centralize the access to all of our community services. So right now we deliver community-based services out of dozens and dozens of different locations and all of these teams do their own intake and it makes system navigation very difficult … So instead of having 35 different phone numbers that you can call for help, we're going to have one. And whoever answers the phone is going to have full understanding of all of our programs and all of our services so that they can schedule you immediately into the right program for you"* (P6118). *"Because I think the key thing for intermediate care – and we can work on it within intermediate care first and then try to expand to the rest of the system – is concepts like a single point of access, a single care plan, a single assessment tool, which are integration factors" *(P8114).

###  Trade-offs in Defining Populations

 Two trade-offs were identified in defining populations. First, programs must make a trade-off between over-including and over-excluding patients – the *sensitivity-specificity trade-off*. Participants clearly described the hazards of both exclusivity and inclusivity, and discussed their challenges in negotiating the balance between them. While some suggested that maintenance of strong, specific criteria inevitably resulted in exclusion of patients with atypical needs, others noted the benefits of defining clear, specific clusters of patient need, noting that earmarking capacity for each cluster improves efficiency and effectiveness of care. Second, health system planners face a trade-off between designating too few programs (resulting in poorly-tailored services for diffuse populations) and too many (resulting in difficulty matching inflexible capacity to variable demand) – the *carve-out trade-off*. We found some consensus that carving out specialized services worked better where patient volumes were higher: The higher patient volume, the greater likelihood of maintaining steady demand for each type of service.

###  Trade-offs in Defining Capacity

 Participants also discussed the trade-off between too much flexibility in deployment of capacity and too little – the *bespoke trade-off.* While flexibility can enable customization to meet the needs of individual patients – promoting person-centred care – overly fluid resources may easily be deployed inefficiently. Participants suggested that, where possible, designing services around cohorts of patients with similar needs is more cost-effective than an individualized approach.

 The considerations involved in defining population and capacity interact: If a population is to be defined broadly, then the capacity assigned to that population must be defined either flexibly (fluid resources) or very generously, to accommodate a shifting “fuzzy set” of needs. Conversely, if a program intended to serve a broad population defines its capacity narrowly, many patients will *de facto* be excluded because “we don’t do that here.”

###  Trade-offs in Defining Process

 Finally, when discussing the challenges of finding appropriate care for patients, participants identified a trade-off between eliminating bottlenecks early vs. late in the process – the *bottleneck trade-off. *Participants commonly noted that a *sorting model,* in which referring providers learn each program’s eligibility criteria and contact one after another until their patient is accepted, creates bottlenecks at the point of referral. Several suggested moving to what could be called *funnel* models, in which a receiving program with broad scope takes responsibility for placing each referred patent in the most appropriate sub-program and/or providing direct service to patients for whom no suitable program exists. However, the convergence of diverse patients on one large funnel can create a bottleneck at the point of assessment, and adds a needless extra step for those patients who could easily have been sent directly to the right program. There was no apparent consensus as to how numerous or broad the funnels should be or which patients should use them.

 We found striking similarities in experiences and perspectives across regions, irrespective of their size or organizational structure; however, informants from regions that were in the throes of restructuring provided more observations relevant to population definition. Hospital leaders and medical directors contributed the most on this topic; lower-level managers and QI personnel the least. Although participants expressed diverse opinions on the desirability of narrow/rigid vs. broad/flexible ways of defining populations, the issue did not appear to be highly polarized; that is, perspectives varied not only between but within groups (eg, sites, programs), and several participants said they could “see both sides” of a trade-off. However, we noted that narrow/rigid vs. broad/flexible models tended to be advocated in relation to *different patient populations*. The former were most commonly recommended for surgical patients (especially elective surgery, which lends itself to pathways and predictability) or conditions defined by an acute event, such as stroke. The latter were most commonly recommended for heterogeneous populations resistant to sub-grouping, especially those characterized by multiple, continuing needs and diverse patterns of comorbidity (eg, frail older adults).

## Discussion

 Across the sample of regions, participants reported the same three strategies for addressing population–capacity misalignment, and in the same order of prevalence: mutual accommodation, capacity (re)allocation, and population (re)definition. It would seem logical, in light of the population-capacity-process model,^9^ for systems to first define populations on the basis of need, then allocate sufficient capacity to each population, and only then promote mutual accommodation to handle the few inevitable exceptions. Instead, we observed the reverse pattern: Process solutions were the default option; capacity-related reforms were undertaken sometimes (but not as often as needed); and no region had taken a systematic, comprehensive approach to defining populations.

 This pattern likely reflects the relative level of difficulty of the three strategies. Mutual accommodation is a case-by-case approach that operational managers can undertake independently on a daily basis, with no major structural, policy, or budgetary implications. Capacity allocation, in contrast, entails system-level change, which must be pursued by strategic decision-makers on a longer planning horizon. Population redefinition requires that systems actually be *redesigned*, an even more complex and disruptive prospect. Furthermore, although we identified a simple principle to guide the definition of populations – namely, group patients on the basis of clusters of need – the task of identifying such clusters is not so simple in practice. Health system planners confront a host of trade-offs as they struggle to define populations and the associated service models in ways that are neither too narrow and rigid nor too broad and flexible. Yet, without effective redefinition of populations, population–capacity misalignment seems guaranteed to persist, with associated patient risk and waste to the system. Mutual accommodation is a classic example of the ineffective application of non-system-level solutions to system-level problems; capacity (re)allocation offers a superior solution, but will yield limited benefit unless program boundaries are aligned with clusters of need. How, then, should planners approach the challenging enterprise of population redefinition?

 If our findings are to be converted into practical guidance for system design, it is necessary to derive a general principle for managing the identified trade-offs. No participant articulated such a principle. However, the finding that narrow/rigid models tended to be recommended for homogeneous populations and broad/flexible models for heterogeneous models provides an important clue: It suggests that the optimal balance point in each of the trade-offs may depend on the nature and distribution of needs in the target population. On this basis, we advance the following supposition: *The greater the separability of clusters of need, the greater precision can and should be applied when defining population, capacity, and process*.

 Although the separability of clusters of need is a continuum, not a binary construct, for the sake of clarity we will define two ideal-types of populations: those in which clusters of need are clearly *separable* (S populations) and those in which they are unavoidably *fused *(F populations). Findings suggest that the two types of populations demand different approaches to system design: S populations, which can be divided into homogeneous, needs-based groups with high coverage and low overlap, are best served by relatively narrow/rigid definitions; F populations, which feature diverse, overlapping constellations of need, require broad/flexible definitions. Accordingly, for S populations, it is most efficient to apply what we call the three Ss of system design: *segmentation* (create a separate program or stream for each cluster), *specified services *(provide a fixed set of services within each program or stream), and *sorting rules *(define policies to manage common types of exceptions). F populations, in contrast, demand the three Fs of system design: *fuzzy sets* (defining clusters of needs in general terms, avoiding arbitrary or excessively specific criteria), *fluid resources* (facilitating add-ons to core services for rapid tailoring), and/or *funnels *(making the initial ‘gate’ to services as wide as possible, with the onus on the receiving program). Figure offers a visual representation of service models for S vs. F needs.

**Figure:**
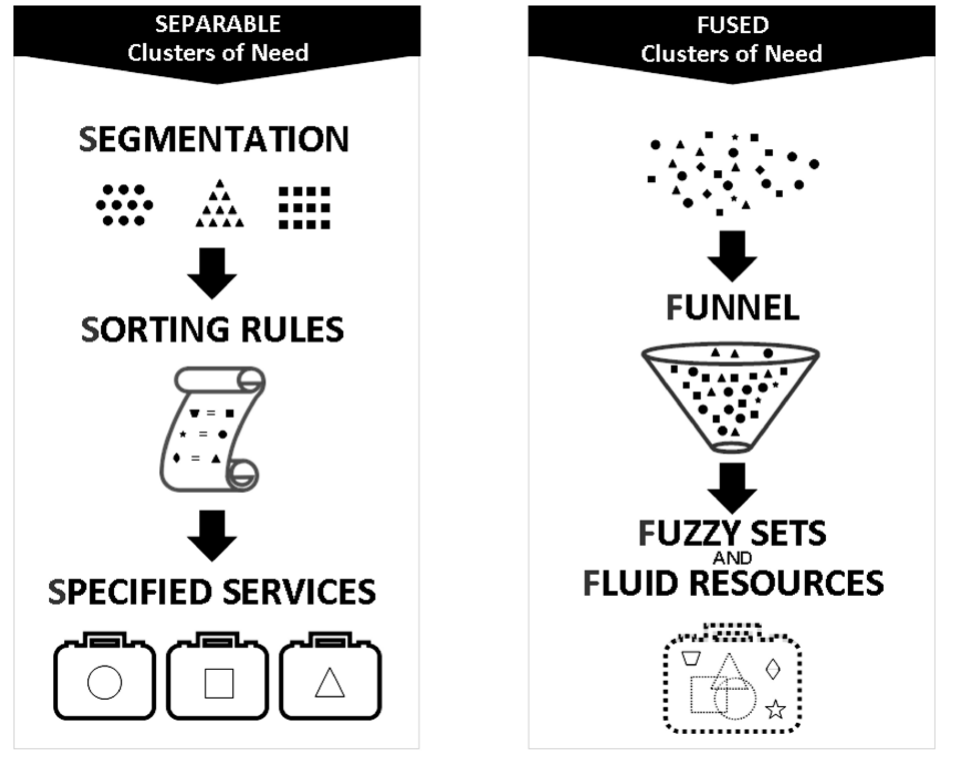
Models of System Design for Separable vs. Fused Clusters of Need.

 It may sometimes be possible to identify a cluster of “S” needs in a population that otherwise has “F” features (eg, for gastric banding surgery in the bariatric population). Under such circumstances, it would seem desirable to use an S model for the well-defined, time-limited needs, while ensuring that an F model exists to manage the open-ended needs (cf. Christensen et al, on differentiation of services for different categories of need).^34^

 The three Ss of system design are highly congruent with the flow literature. S strategies aim to reduce within-program variability in patient needs (segmentation) and care activities (specified services), and to increase the speed of patient assignment (sorting rules); in this way they address both of the domains identified by the Theory of Swift, Even Flow.^15^ The strategy of segmentation/streaming, which derives from queuing theory, underpins many flow interventions,^35,36^ and the carve-out trade-off has been discussed from an operations research or process engineering perspective.^18^ However, this literature does not appear to address the issue of cluster-resistant populations. While some research suggests that process engineering methodologies such as Lean are most likely to achieve gains when applied to populations with relatively narrow, homogeneous sets of needs,^37^ it is unclear to what extent such methodologies (and the theories that inform them) are truly applicable to complex populations. Meanwhile the growing literature on centralized/pooled waiting lists offers some support for the idea that narrow-funnel models can improve access to a defined service, but this literature has not addressed broad-funnel models.^38,39^

 The three Fs of system design are congruent with leading models of integrated care for seniors and persons with continuing care needs.^20,40^ Two key features of such models are a single point of entry with a common assessment process (funnel) and a shared resource envelope across sectors (fluid resources).^41^ The integration literature recognizes that not all patients require integrated care, nor is it cost-effective to spread high-intensity services beyond their intended population; thus there is a need to draw program boundaries – and an accompanying risk of creating gaps, silos, and fragmentation.^42^ However, it is essential that integrated models be available to patients who require them; otherwise, the burden of complex, open-ended needs will continue to fall on services ill-equipped to meet them, notably EDs.^43^

 The overall principle of defining programs around identified clusters of need is congruent with leading work on delivery-system redesign, such as the Porter and Lee proposal for creation of Integrated Practice Units.^44^ Our research builds on this conceptual foundation by proposing that health system planners should first examine how tightly or loosely needs are clustered within the population(s) to be served. Although there are several statistical methods for analyzing clustering of individuals or characteristics,^45-49^ research to date has not compared populations in terms of separability of clusters. Studies of older adults with multimorbidity have yielded conflicting results as to whether this population can be accurately subcategorized, and, if so, what these categories might be.^50-52^ Further research is required in this area. A key challenge will be collecting data to support such analyses, as most administrative datasets include data on patient characteristics, diagnoses, and interventions received, but not necessarily *needs. *

 Our finding that all regions showed evidence of population–capacity misalignment supports Kreindler’s^23^ conjecture that this problem is not unique to one poorly performing system but afflicts health systems in general. In contrast to Kreindler,^23^ we did not observe severe, intractable conflict of the “Your Order Is My Chaos” variety. Most regions appeared to mitigate conflict through intensive processes of mutual accommodation; in Kreindler’s earlier study, the region examined appeared to lack sufficient central authority to enforce such processes, and allowed conflict to persist unmanaged. We suggest that intergroup conflict is merely one potential symptom of population–capacity misalignment, and that the latter, not the former, should be considered the hallmark of this “paradox of patient flow.”

 In summary, our findings suggest that population–capacity misalignment might best be tackled through the following process: First, assess whether clusters of need are S or F; second, design services around clusters of need using S or F models as appropriate; third, assign capacity according to the observed distribution of needs. Any unforeseeable configurations of need should then – and only then – be dealt with through mutual accommodation.

###  Limitations

 This study has a number of limitations. None of the study sites had conquered their patient flow problems, and all were in Western Canada. In a recent 11-country comparison, Canada ranked last on timeliness and below average on coordination of care.^53^ Population­–capacity misalignment may be particularly severe in the fragmented Canadian system, in which policy-makers have typically lacked strong mechanisms to influence the delivery of care despite holding the funding envelope.^54^ There is a need to extend research on population–capacity (mis)alignment to higher-performing countries. However, as ED crowding is an international problem^10^ and patterns of patient need are not country-specific, we believe that the identified principles for system design will prove transferable beyond Canada.

 Our sample was restricted to management: While many participants were also practicing clinicians, we did not obtain perspectives of frontline providers or service users; incorporation of these perspectives would enrich future work. There is also a possibility of respondent bias: Although we recruited a diverse sample of individuals with strategic or operational responsibility for flow across the continuum of care, those who chose not to participate may have had differing perspectives. We also note that only a portion of each interview was focused on system design, and we did not explicitly ask participants, “Should we change the way we think about defining populations and, if so, what are the correct principles for doing so?” However, as the interview questions and probes afforded multiple opportunities for participants to address population redefinition, we believe that the relative paucity of data on this strategy reflected an actual gap in system-design thinking/practice within our sample, rather than a gap in the interview guide.

 While an exploratory, interpretive approach enabled preliminary theory generation, the emerging theory must be tested through further research, and with systems exhibiting a greater range of performance. We were able to identify principles for system design, but not to assess the effectiveness or feasibility of any particular way of operationalizing these principles (eg, models advocated by certain participants). Consequently, our findings cannot be used to recommend specific system design initiatives. The breadth and flexibility of study design did, however, facilitate generation of novel concepts and hypotheses, which we offer for further exploration.

 Finally, in focusing on the underexplored issue of population–capacity misalignment, we do not mean to imply that this is the only possible cause of flow problems, much less that adding capacity is always part of the solution. Poor performance may instead reflect inefficient use of capacity,^18^ or weak execution and coordination of improvement initiatives.^8^ Only local investigation can ascertain the most important contributing factors within a particular system.^10^

## Conclusion

 Population–capacity misalignment will continue to adversely affect patient flow until health services are redesigned to match observed clusters of population need. Our findings suggest that system redesign should be guided by the following fundamental principle: Select S or F service models according to whether clusters of need are “separable” or “fused.” However, we found little evidence that this principle was being applied in practice. Instead, health systems may wastefully provide S populations with F models, or, more commonly, attempt to serve F populations with a miscellany of S models, leaving providers with the slow, frustrating task of reinventing the system for each atypical patient. Robust F models could effectively meet the needs of heterogeneous populations, such as the “fuzzy set” of community-dwelling older adults with continuing care needs. The consequence of failure to develop such models can be observed in the ED, which sees an ever-growing volume of complex patients whose needs are not being met elsewhere.^43^ As a result, a service originally intended to provide *rapid* care instead becomes the ultimate F model: a giant funnel offering unlimited resources to a population with no eligibility criteria. Thus, until we invest in F models where they are most efficient and appropriate, we will continue to use them where they are most inefficient and inappropriate.

## Acknowledgements

 This study was supported by a Canadian Institutes of Health Research – Partnership for Health Systems Improvement grant (PHE-141802), with partnership funding from the Michael Smith Foundation for Health Research, Alberta Innovates, Saskatchewan Health Research Foundation and Research Manitoba. We are grateful to Sarah Bowen for her invaluable help with the process of transforming a report chapter into an article. We also thank the full WeCanFlow researcher/decision-maker team for their helpful feedback, Reena Kreindler for her editorial advice, and Leah Nicholson for manuscript preparation assistance.

## Ethical issues

 This study was approved by the University of Manitoba Health Research Ethics Board, UBC Providence Health Care Research Ethics Board, University of Calgary Conjoint Health Research Ethics Board, and University of Saskatoon Behavioural Research Ethics Board. Participants provided free and informed consent in writing.

## Competing interests

 Authors declare that they have no competing interests.

## Authors’ contributions

 SK conceived and designed the study; SK, SH, SW, KJ, SM, and MB conducted interviews; SK and ZA conducted data analysis; all authors were involved in interpretation of findings. SK drafted the manuscript, and all authors provided critical review and feedback.

## References

[1] Institute of Medicine. Crossing the Quality Chasm: A New Health System for the 21st Century. Vol 323. Washington, DC: National Academies Press; 2001. 25057539

[2] Asplin BR, Magid DJ, Rhodes KV, Solberg LI, Lurie N, Camargo CA Jr (2003). A conceptual model of emergency department crowding. Ann Emerg Med.

[3] Crawford K, Morphet J, Jones T, Innes K, Griffiths D, Williams A (2014). Initiatives to reduce overcrowding and access block in Australian emergency departments: a literature review. Collegian.

[4] Chan SS, Cheung NK, Graham CA, Rainer TH (2015). Strategies and solutions to alleviate access block and overcrowding in emergency departments. Hong Kong Med J.

[5] Winasti W, Elkhuizen S, Berrevoets L, van Merode G, Berden H (2018). Inpatient flow management: a systematic review. Int J Health Care Qual Assur.

[6] de Grood J, Bota M, Villa-Roel C, et al (2012). Overview of interventions to mitigate emergency department overcrowding.

[7] De Freitas L, Goodacre S, O’Hara R, Thokala P, Hariharan S (2018). Interventions to improve patient flow in emergency departments: an umbrella review. Emerg Med J.

[8] Chang AM, Cohen DJ, Lin A (2018). Hospital strategies for reducing emergency department crowding: a mixed-methods study. Ann Emerg Med.

[9] Kreindler SA (2017). Six ways not to improve patient flow: a qualitative study. BMJ Qual Saf.

[10] Morley C, Unwin M, Peterson GM, Stankovich J, Kinsman L (2018). Emergency department crowding: a systematic review of causes, consequences and solutions. PLoS One.

[11] Kolker A (2013). Interdependency of hospital departments and hospital-wide patient flows.

[12] Sullivan CM, Staib A, Flores J (2014). Aiming to be NEAT: safely improving and sustaining access to emergency care in a tertiary referral hospital. Aust Health Rev.

[13] Weber EJ, Mason S, Carter A, Hew RL (2011). Emptying the corridors of shame: organizational lessons from England’s 4-hour emergency throughput target. Ann Emerg Med.

[14] Goldratt EM, Cox J. The Goal: A Theory of Constraints. Barrington, MA: North River Press; 1984.

[15] Schmenner RW, Swink ML (1998). On theory in operations management. J Oper Manag.

[16] Jensen K, Mayer TA, Welch S, Haraden C. Leadership for Smooth Patient Flow: Improved Outcomes, Improved Service, Improved Bottom Line. Chicago, IL: Health Administration Press with the Institute for Healthcare Improvement; 2007.

[17] Kreindler SA (2008). Watching your wait: evidence-informed strategies for reducing health care wait times. Qual Manag Health Care.

[18] Litvak E, Long MC (2000). Cost and quality under managed care: irreconcilable differences?. Am J Manag Care.

[19] Radnor ZJ, Holweg M, Waring J (2012). Lean in healthcare: the unfilled promise?. Soc Sci Med.

[20] Hollander MJ, Prince MJ (2008). Organizing healthcare delivery systems for persons with ongoing care needs and their families: a best practices framework. Healthc Q.

[21] Wagner EH, Austin BT, Davis C, Hindmarsh M, Schaefer J, Bonomi A (2001). Improving chronic illness care: translating evidence into action. Health Aff (Millwood).

[22] Grembowski D, Schaefer J, Johnson KE (2014). A conceptual model of the role of complexity in the care of patients with multiple chronic conditions. Med Care.

[23] Kreindler SA (2017). The three paradoxes of patient flow: an explanatory case study. BMC Health Serv Res.

[24] Anwar MR. A Realist Analysis of Streaming Interventions in Emergency Departments [thesis]. Winnipeg: University of Manitoba; 2019.

[25] Roemeling O, Ahaus K, van Zanten F, Land M, Wennekes P (2019). How improving access times had unforeseen consequences: a case study in a Dutch hospital. BMJ Open.

[26] Birch S, Mason T, Sutton M, Whittaker W (2013). Not enough doctors or not enough needs? refocusing health workforce planning from providers and services to populations and needs. J Health Serv Res Policy.

[27] Pennel CL, McLeroy KR, Burdine JN, Matarrita-Cascante D (2015). Nonprofit hospitals’ approach to community health needs assessment. Am J Public Health.

[28] Aoun S, Pennebaker D, Wood C (2004). Assessing population need for mental health care: a review of approaches and predictors. Ment Health Serv Res.

[29] Santibáñez P, Bekiou G, Yip K (2009). Fraser Health uses mathematical programming to plan its inpatient hospital network. INFORMS J Appl Anal.

[30] Olafson K, Ramsey C, Yogendran M (2015). Surge capacity: analysis of census fluctuations to estimate the number of intensive care unit beds needed. Health Serv Res.

[31] Cramer GR, Singh SR, Flaherty S, Young GJ (2017). The progress of US hospitals in addressing community health needs. Am J Public Health.

[32] Latest update: Structural profile of public health in Canada. National Collaborating Centre for Healthy Public Policy website. https://ncchpp.ca/710/Structural_Profile_of_Public_Health_in_Canada.ccnpps. Accessed November 5, 2019.

[33] Strauss A, Corbin J. Basics of Qualitative Research: Grounded Theory Procedures and Techniques. 2nd ed. London: SAGE Publications; 1998.

[34] Christensen CM, Grossman JH, Hwang J. The Innovator’s Prescription: A Disruptive Solution for Health Care. New York: McGraw-Hill Education; 2009.

[35] Rothkopf MH, Rech P (1987). Perspectives on queues: combining queues is not always beneficial. Oper Res.

[36] Whitt W (1999). Partitioning customers into service groups. Manage Sci.

[37] Mazzocato P, Thor J, Bäckman U (2014). Complexity complicates lean: lessons from seven emergency services. J Health Organ Manag.

[38] Damani Z, Conner-Spady B, Nash T, Tom Stelfox H, Noseworthy TW, Marshall DA (2017). What is the influence of single-entry models on access to elective surgical procedures? a systematic review. BMJ Open.

[39] Breton M, Smithman MA, Sasseville M (2020). How the design and implementation of centralized waiting lists influence their use and effect on access to healthcare - a realist review. Health Policy.

[40] Kodner DL, Spreeuwenberg C (2002). Integrated care: meaning, logic, applications, and implications--a discussion paper. Int J Integr Care.

[41] MacAdam M. Frameworks of Integrated Care for the Elderly. https://brainxchange.ca/Public/Files/Primary-Care/HQPC/Care-of-the-Eldery-integrate-care.aspx. Published April, 2008. Accessed November 7, 2019.

[42] Leutz WN (1999). Five laws for integrating medical and social services: lessons from the United States and the United Kingdom. Milbank Q.

[43] Kreindler SA, Cui Y, Metge CJ, Raynard M (2016). Patient characteristics associated with longer emergency department stay: a rapid review. Emerg Med J.

[44] Porter ME, Lee TH (2013). The strategy that will fix health care. Harv Bus Rev.

[45] Doupe M, Fransoo R, Chateau D, et al. Population Aging and the Continuum of Older Adult Care in Manitoba. Winnipeg: Manitoba Centre for Health Policy; 2011.

[46] Liu LK, Guo CY, Lee WJ (2017). Subtypes of physical frailty: latent class analysis and associations with clinical characteristics and outcomes. Sci Rep.

[47] Simo B, Bamvita JM, Caron J, Fleury MJ (2018). Patterns of health care service utilization by individuals with mental health problems: a predictive cluster analysis. Psychiatr Q.

[48] Subbe CP, Goulden N, Mawdsley K, Smith R (2017). Anticipating care needs of patients after discharge from hospital: frail and elderly patients without physiological abnormality on day of admission are more likely to require social services input. Eur J Intern Med.

[49] Yan S, Kwan YH, Tan CS, Thumboo J, Low LL (2018). A systematic review of the clinical application of data-driven population segmentation analysis. BMC Med Res Methodol.

[50] Foguet-Boreu Q, Violán C, Rodriguez-Blanco T (2015). Multimorbidity patterns in elderly primary health care patients in a South Mediterranean European region: a cluster analysis. PLoS One.

[51] Larsen FB, Pedersen MH, Friis K, Glümer C, Lasgaard M (2017). A latent class analysis of multimorbidity and the relationship to socio-demographic factors and health-related quality of life A national population-based study of 162,283 Danish adults. PLoS One.

[52] Whitson HE, Johnson KS, Sloane R (2016). Identifying patterns of multimorbidity in older Americans: application of latent class analysis. J Am Geriatr Soc.

[53] Schneider EC, Sarnak DO, Squires D, Shah A, Doty MM. Mirror, Mirror 2017: International Comparison Reflects Flaws and Opportunities for Better U.S. Health Care. New York: The Commonwealth Fund; 2017.

[54] Martin D, Miller AP, Quesnel-Vallée A, Caron NR, Vissandjée B, Marchildon GP (2018). Canada’s universal health-care system: achieving its potential. Lancet.

